# Detection of Quinoline in *G. boninense*-Infected Plants Using Functionalized Multi-Walled Carbon Nanotubes: A Field Study

**DOI:** 10.3390/s17071538

**Published:** 2017-07-01

**Authors:** Fowotade Sulayman Akanbi, Nor Azah Yusof, Jaafar Abdullah, Yusran Sulaiman, Roozbeh Hushiarian

**Affiliations:** 1 Department of Chemistry, Faculty of Science, Universiti Putra Malaysia, Serdang, Selangor 43400, Malaysia; fowotades14@gmail.com (F.S.A.); jafar@upm.edu.my (J.A.); yusran@upm.edu.my (Y.S.); 2 Department of Science Laboratory Technology, Hussaini Adamu Federal Polytechnic, A2 Kazaure, Nigeria; 3 Institute of Advanced Technology, Universiti Putra Malaysia, Serdang, Selangor 43400, Malaysia; 4 La Trobe Institute for Molecular Science, La Trobe University, Victoria 3086, Australia

**Keywords:** electrochemical sensor, screen-printed carbon electrode, multi-walled carbon nanotubes, quinoline, *Ganoderma boninense*

## Abstract

Carbon nanotubes (CNTs) reinforced with gold nanoparticles (AuNPs) and chitosan nanoparticles (CTSNPs) were anchored on a screen-printed electrode to fabricate a multi-walled structure for the detection of quinoline. The surface morphology of the nanocomposites and the modified electrode was examined by an ultra-high resolution field emission scanning electron microscope (FESEM), and Fourier-transform infrared (FT-IR) spectroscopy was used to confirm the presence of specific functional groups on the multi-walled carbon nanotubes MWCNTs. Cyclic voltammetry (CV) and linear sweep voltammetry (LSV) were used to monitor the layer-by-layer assembly of ultra-thin films of nanocomposites on the surface of the electrode and other electrochemical characterizations. Under optimized conditions, the novel sensor displayed outstanding electrochemical reactivity towards the electro-oxidation of quinoline. The linear range was fixed between 0.0004 and 1.0 μM, with a limit of detection (LOD) of 3.75 nM. The fabricated electrode exhibited high stability with excellent sensitivity and selectivity, specifically attributable to the salient characteristics of AuNPs, CTSNPs, and MWCNTs and the synergistic inter-relationship between them. The newly developed electrode was tested in the field. The Ipa increased with an increase in the amount of quinoline solution added, and the peak potential deviated minimally, depicting the real capability of the newly fabricated electrode.

## 1. Introduction

*Ganoderma boninense (G. boninense)* is a necrotroph well-entrenched in oil palm plantations worldwide [1,2,3]. As well as *G. boninense*, other species causing the disease are *G. zonatum,* and *G. miniatotinctum* [1,2,4]. Southeast Asian countries, particularly Malaysia and Indonesia, are reported to have struggled with the economic impact of *G. boninense* on oil palm since 1928 [4] due to the lack of an effective method of detection at the onset of infection. This lethal fungus is also referred to as white rot fungus because it reacts with lignin, a shield against microbial attack in the oil palms, to produce white coloured cellulose further powering the fungus. The biodegradation of lignin is a secondary metabolic reaction, which indicates the disease pathway [5]. On attacking the palm trees, an arsenal of secondary metabolites are released to combat the incursion of the fungus [1,6]. One of these metabolites, produced within 24 h of a *G. boninense* attack, is quinoline as reported by [3]. Quinoline belongs to the alkaloids group of secondary metabolites [7,8] and is derived from tryptophan, an amino acid based precursor. 

Alkaloids are a large division of plant-related chemicals which may be toxic, medicinal, nutritive, cosmetic-based, or pathogenic to plants. They are members of a group of secondary metabolites called phytoalexins, released from plants in defensive response to pathogenic attacks from fungi, bacteria, or other stressors [8]. The resistant and susceptible host plants possess equal levels of phytoalexin at different rates. The production time of this group of secondary metabolites at the point of infection largely determines their efficiency [9]. *G. boninense* often affects the lower stem region, causing basal stem rot (BSR) disease which extends to the entire root system [10,11] leading to complete deterioration. BSR diseases cut across the various ages of oil palms both young and old, although the highest level of severity has been observed in old trees, especially in Indonesia and Malaysia [2]. It has also been reported that ex-coconut cultivated land oil palms are more susceptible to the disease, in contrast to the more resistant palms from ex-rubber plantations [2]. This destruction of palms has precipitated a massive loss of foreign exchange in the palm oil sector of a number of countries. Losses to the tune of over three hundred million US dollars and over nine hundred million US dollars in Malaysia and Indonesia, respectively, have been reported [2,3,10]. The cost of each palm tree killed by BSR has been estimated to be as high as eight hundred US dollars. Thus, there is a dire need to curtail further destruction of palms by *G. boninense* attack and early detection of BSR is critical. 

In response to demand from the sector, scientists have been working on a range of methods [2], but to date, none have been embraced by the industry. Some methods have included polymerase chain reaction (PCR) amplification [12,13,14] but have encountered challenges of gene bank aberrations resulting from sequencing [2,13,15]. Other recent methods have used electrochemical sensors and have involved the complex process of extracting DNA from the infected part of a tree [16,17,18,19]. The disease has also been detected via hyperspectral data and imagery techniques [2,20,21] with the attendant problems of tying to observe palm leaves coupled at the top of palm canopies. Several attempts employed an enzyme-linked immunosorbent assay (ELISA) to detect BSR [2,10].

In the past few years, there have been substantial advances in electroanalytical approaches with the emergence of nanotechnology and disposable electrode technology. The latter facilitated the development of screen printed electrodes (SPEs), which are small, disposable, portable, selective, sensitive, and inexpensive with their negligible power consumption when compared with earlier heavy and complicated electrodes. SPE is a three electrodes system, cemented on a support that is chemically inactive. While electrochemical processes take place on the working electrode, the reference and counter electrodes activate the circuit [22]. Nanotechnology allows for the application of objects in a nano size range [23] and this study focuses specifically on nanotubes and nanoparticles. Multi-walled carbon nanotubes (MWCNTs) possess high surface to volume ratio but their large surface area is extremely limited due to their poor dispersion in common solvents. This in turn affects their gas adsorptive, toxic, catalytic, electric, thermal, and mechanical capacities and limits their applications [24]. Fortunately, MWCNTs can be activated with the aid of oxidizing agents to promote their dispersion in common solvents and ignite their inherent qualities. This activation enhances the surface area enlargement and also introduces carboxylic (-COOH) groups which serve as linkages to other reactive species [25]. Additionally, MWCNTs with a high level of crystallinity act to restrict oxidative chemical agents [24]. Activated MWCNTs can be juxtaposed with other nano-sized materials to create nanocomposites with improved quality in such applications as nanosensors in electrochemical detection [26]. Chitosan (CTS) is one example, being a non-artificial polymer capable of dissolving in aqueous acidic solvents. It bears a positive charge while in solution, thus easily attaching to any substance with a negative charge, and it also displays good film formation, zero toxicity, high mechanical strength, and excellent water permeability [27]. Gold nanoparticles (AuNPs) are another kind of nanomaterial known for their catalytic ability and particle size-dependent use [17,19]. Modification of carbon nanotubes (CNTs) with CTS and AuNPs has been successfully carried out and reported [28]. 

**Figure 1:**
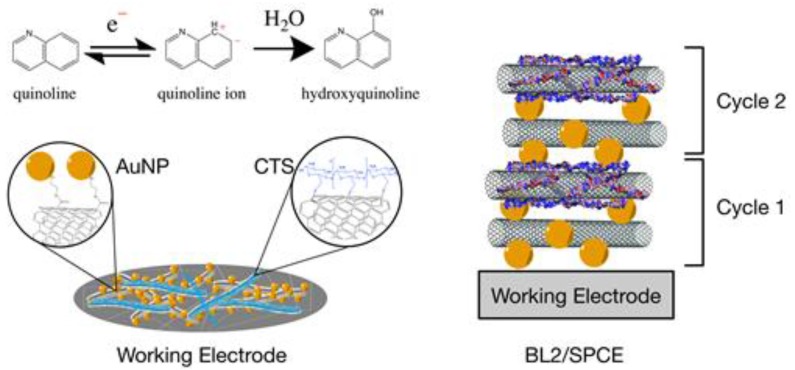
Schematic diagram of the modified electrode and the reaction of quinoline on the surface of the electrode.

The present study took an alternative electrochemical sensor pathway as its approach to the early detection of BSR disease in oil palms [29]. Figure 1 demonstrates the fabricated electrode surface and the reaction of quinoline on the surface of the working electrode. Two sets of nanocomposites were prepared. Activated multi-walled carbon nanotube-chitosan (aMWCNT-CTS) and activated multi-walled carbon nanotube-gold nanoparticles (aMWCNT-AuNPs) were employed to modify the surface of a disposable screen printed carbon electrode (SPCE), using a layer-by-layer assembly method. The nanomaterials were grafted to the surface of the activated MWCNTs to produce the nanocomposites. The characterization of the fabricated electrode was achieved using high resolution field emission scanning electron microscopy (FESEM) and electrochemical approaches. It was discovered that the ultra-thin films of the nanocomposites were well layered on the SPCEs and were capable of detecting quinoline in oil palm leaf extracts. The modified electrode gave better sensing and higher sensitivity than ordinary SPCE and aMWCNT/SPCEs, potentially offering a new option for constructing an effective electrochemical sensor.

## 2. Materials and Methods

### 2.1. Reagents

Quinoline (97 wt % in water), multi-walled carbon nanotubes (90±%, 110–170 nm in diameter and 5–9 μm in length), gold(III) chloride trihydrate (≥99.9%), sodium citrate dehydrate (≥99%), and low molecular chitosan were all supplied by Sigma-Aldrich (St. Louis, MO, USA). Methanol (80 wt %), ethanol (99.8%), and sulphuric acid (95–97%) stock solutions were purchased from Friendemann Schmidt (Parkwood, Australia). Iron (III) trioxonitrate (V) non hydrate was procured from Fluka (Durban, South Africa). Potassium chloride and potassium hydroxide were bought from R & M chemicals (Edmonton, AB, Canada). Deionized (DI) water was used for every reaction and cleansing of reaction vessels. Different buffer solutions of 0.2 M with approximately the same pH were prepared adjusting mixed acid solutions comprising glacial acetic acid, citric acid, phosphate with 2N NaOH solution, while mixed basic solutions of carbonate and borate comprised 1N HCl solution.

### 2.2. Instruments

A digital balance used to accurately measure the weight of various solids was supplied by A & D Company Ltd. (Tokyo, Japan). A digitalized pH-meter, purchased from Thermo Fisher Scientific Company (Chicago, IL, USA), was employed in the adjustment of pH values of prepared buffer solutions, acting as supporting electrolytes in the voltammetric experiments. Ultrasonic cleaner (power sonic 420), purchased from Hwashin Technology Company (Seoul, Korea), was used to execute sonication of nanocomposites. A Kubato bench top centrifuge was utilized for washing samples and was obtained from Kubato Manufacturing Company (Osaka, Japan). Cyclic voltammetry (CV) and linear sweep voltammetry (LSV) measurements were executed using μStat 8000, DropSens potentiostat (Asturias, Spain). Screen-printed carbon electrodes were obtained from MIMOS (Bukit Jalil, Malaysia) and comprised a working (4 mm diameter) electrode made of carbon, counter electrode made of platinum, and reference electrode made of silver. Fourier-transform infrared (FT-IR) spectroscopy aided the identification of functionality groups attached to the surface of activated carbon nanotubes (CNTs) and was carried out on a Nicolet 6700 FT-IR spectrometer (Waltham, MA, USA). An ultra-high resolution field emission scanning electron microscope (FESEM), Nova Nanosem 230, FEI (Oregon, OR, USA) was utilized to observe the facial morphology of various types of CNTs and modified electrodes. Particle size analysis of synthesized gold nanoparticles (AuNPs) was performed on a Malvern nano S. Nano sizer (Enigma Business Park, UK). 

### 2.3. Preparation of Modified Electrode

Based on the method of Manso and team [30] with a minor modification, 49.3 nm AuNPs was synthesized in solution form. Then, 10 mg low molecular weight chitosan was dissolved in 100 mL DI water to give 0.01% chitosan nanoparticle (CTSNP) solution. The activation of as-purchased MWCNT was actualized by modifying the technique described in the literature [31]. The present study made use of the combination of potassium chloride, potassium hydroxide, and iron (iii) trioxonitrate(v) nonahydrate rather than the potassium tetraoxoferrate (vi) utilized by Zhang and Xu.

The AuNPs and CTSNPs were then successfully grafted on to the activated MWCNTs (aMWCNTs) via sonication and centrifugation to obtain the nanocomposites [25], namely, gold nanoparticles-activated multi-walled carbon nanotubes (AuNPs-aMWCNTs) and chitosan nanoparticles-activated multi-walled carbon nanotubes (CTSNPs-aMWCNTs). The synthesis of these nanocomposites followed a protocol from Zheng and co-workers [32] with slight modifications. The main fabrication of the SPCE was actualized using layer-by-layer (LBL) assembly. The assembly approach was achieved by alternately immersing the SPCE in nanoparticle dispersion, DI water, nanoparticle dispersion, and DI water. The immersion time in DI water was 2 min while 5 min was spent in each nanoparticle dispersion medium. The bare SPCE was thus coated by ultra-thin bilayers of AuNPs-aMWCNTs and CTSNPs-aMWCNTs, resulting in a complete cycle deposition. The fabricated electrode is AuNPs-aMWCNTs/CTSNPs-aMWCNTs/SPCE. For simplicity, this electrode will be referred to as BL1/SPCE (one bilayer of nanocomposites on SPCE surface). The cycle was continued to produce other electrodes with the required number of nanoparticle modifier bilayers. After successful completion of the required number of cycles, the resultant electrode was oven dried at 500 C. The acronym BLn/SPCE will be used in referring to the newly fabricated electrode in subsequent sections, where *n* = 1, 2, 3 and so on.

## 3. Results and Discussions

### 3.1. Surface Morphology of Nanocomposites and Fabricated Bi-Layer Electrode

**Figure 2:**
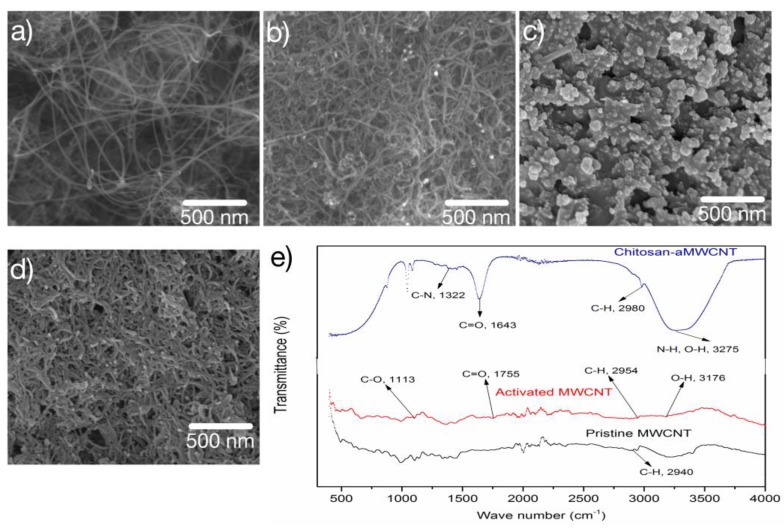
Field emission scanning electron microscope (FESEM) images of (**a**) Pristine multi-walled carbon nanotube (MWCNT), (**b**) gold nanoparticles-activated multi-walled carbon nanotubes (AuNPs—aMWCNT) nanocomposite (**c**) Bare screen printed carbon electrode (SPCE), (**d**) three bilayers of nanocomposites on SPCE surface (BL3/SPCE) and (**e**) Fourier-transform infrared (FT-IR) spectral of Pristine MWCNT, Activated MWCNT (aMWCNT), and Chitosan-aMWCNT.

The morphological structures of MWCNT and AuNPs-aMWCNT composites were observed with the aid of FESEM, as displayed in Figure 2a,b, respectively. Uniform and equivalent tubular arrangement with some voids can be observed (Figure 2a). The tubes are not shortened on activation, although the spaces are closed up to an extent and the surface of pristine-MWCNT is uneven after activation [33] as a result of the attachment of carboxylic (–COOH) functional groups to the surface. The near zero tubular reduction of the aMWCNTs corroborates the nomenclature of the method applied for activating pristine-MWCNTs, known as non-destructive covalent functionalization [31]. The surface of aMWCNT gets craggier with the incorporation of AuNPs (Figure 2b). This high degree of roughness affirms the successful loading of AuNPs on the aMWCNT.

The variously modified bi-layer electrode (BLn/SPCEs) surfaces were assessed by subjecting each electrode to FESEM after the LBL assembly architectural framework that helped to deposit ultra-thin layers of modifiers on its surface. As is shown in Figure 2c,d, the stepped coverage of the SPCE surface is apparent. This is further affirmed by the increase in the roughness of the micrographs in Figure 2d, supporting the increase in the number of bi-layer modifiers or number of cycles through which the electrode surface is immersed in the modifier’s dispersion media. The catalytic AuNPs enhance the rapid transfer of the new electrode’s electron capacity while CTSNPs are responsible for efficient binding potential with the analyte through their functional groups and by improving the aMWCNTs’ mechanical strength. The summative effect of large surface area, increased catalytic capacity, increased strength, and effective binding potential contributed to the overall performance of the newly fabricated electrode. It should be noted that FESEM was used to monitor the coverage of the nanocomposites on the SPCE (Figure 2c,d). The thickness of the layers of the surface modifiers on SPCE, however, was electrochemically monitored using CV, and this is discussed later in the paper. 

The structure of the MWCNTs, activated-MWCNTs, and CTSNPs-aMWCNTs were studied utilizing Fourier-transform infrared (FT-IR) spectroscopy as exhibited in Figure 2e. Feature peaks at wave numbers 3275 cm^−1^, 1643 cm^−1^, and 1322 cm^−1^ equivalent to (–N–H, –O–H), (C=O), and (C–N), respectively, confirmed the realization of CTSNPs-aMWCNTs composites [34,35]. The peak with wave number 2940 cm^−1^ indicates the presence of C-H bonds, a dominating bond in Pristine-MWCNTs while peaks with wave numbers 1755 cm^−1^, 3176 cm^−1^, and 1113 cm^−1^ corresponding to (C=O), (-O-H), and (-C-O), respectively, suggest the carboxylation of the pristine-MWCNTs.

### 3.2. Electrochemical Response of 10 μM Quinoline at Various Electrodes

**Figure 3:**
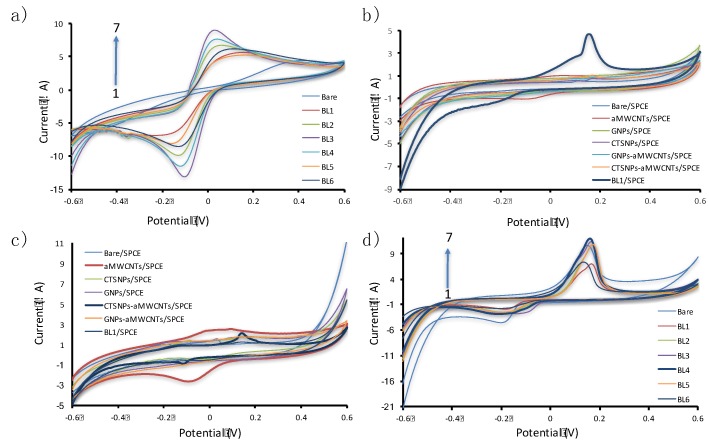
(**a**) Cyclic voltammograms of 1.0 mM K_3_Fe(CN)_6_ and 0.1 M KCl at bare SPCE (1), BL5/SPCE (2), BL1/SPCE (3), BL6/SPCE (4), BL2/SPCE (5), BL3/SPCE (6), and BL4/SPCE (7) at a scan rate of 0.05 V/s. (**b**) Cyclic voltammetry (CV) curves of Bare/SPCE, aMWCNTs/SPCE, gold nanoparticles (AuNPs)/SPCE, chitosan nanoparticles (CTSNPs)/SPCE, AuNPs-aMWCNTs/SPCE, chitosan nanoparticles-activated multi-walled carbon nanotubes (CTSNPs-aMWCNTs)/SPCE BL1/SPCE in 0.2 M citrate buffer solution (CBS) with 10 μM quinoline and (**c**) without quinoline. (**d**) Cyclic voltammograms of 10 μM quinoline at bare/SPCE (1), BL1/SPCE (2), BL2/SPCE (3), BL5/SPCE (4), BL6/SPCE (5), BL3/SPCE (6), and BL4/SPCE/SPCE (7) in 0.2 M CBS at a scan rate of 0.05 V/s. The acronym BLn/SPCE is used to refer to the newly fabricated electrodes, where *n* = 1, 2, 3 layers and so on.

Cyclic voltammograms of 1.0 mM K_3_Fe(CN)_6_ and 0.1M KCl of the bare SPCE (Figure 3a) showed an increase in the signal from bare electrode to four cycles of surface modification and then the signal reduced by further increment of the layers due to the thickness of the modifier acting as a barrier against electron transfer.

The electrochemical efficacy of 10 μM quinoline at bare/SPCE, aMWCNTs/SPCE, AuNPs/SPCE, CTSNPs/SPCE, AuNPs–aMWCNTs/SPCE, CTSNPs-aMWCNTs/SPCE, and CTSNPs-aMWCNTs/AuNPs-aMWCNTs/SPCE (BL1/SPCE) was studied utilizing cyclic voltammetry. Figure 3b reveals the absence of anodic peak current at the bare/SPCE, aMWCNTs/SPCE, AuNPs/SPCE, CTSNPs/SPCE, AuNPs-aMWCNTs/SPCE, and CTSNPs-aMWCNTs/SPCE and presence of a brighter, pin-pointed anodic peak at BL1/SPCE. This observation confirmed the inherent combinatorial qualities of the nanocomposites used as surface modifiers in the fabrication of the new electrode. The peak due to BL1/SPCE is only one of many peaks observable from the SPCE due to its unpredictable sensitivity, and for this reason there were some difficulty in working with SPCEs. This potential-driven electrochemical reaction is reversible. The observable pin-pointed peak could be due to the effect of the combined enhancing traits of the AuNPs and CTSNPs on the aMWCNTs on the surface. In addition, the good sensitivity of the bare SPCE used in fabricating the BL1/SPCE may have contributed to the uniquely observed peak. 

The electrochemical responses on the differently modified electrodes in 0.2 M citrate buffer solution (CBS) (pH = 5.5) without quinoline is displayed in Figure 3c. The curves belonging to aMWCNTs/SPCE and CTSNPs-aMWCNTs/SPCE gave negligible peaks. There is no significant anodic peak current from other curves, corroborating the fact that the oxidation peaks are as a result of the electro-oxidation of quinoline. 

The disappearance of the peak in 10 μM quinoline buffered with 0.2 M CBS may be connected to the environment which may not have been as conductive in the absence of quinoline. For this reason, a supporting electrolyte was required for this study. 

Figure 3d shows the cyclic voltammograms of 10.0 μM quinoline in a buffer medium at variously modified bi-layered electrodes, BLn/SPCEs. The lowest anodic peak current (Ipa) was observed with Bare/SPCE (curve 1) while the highest Ipa was recorded with BL4/SPCE. Each voltammogram consists of two peak currents, namely anodic (Ipa) and cathodic peak currents (Ipc). This justifies the reversibility of the electrochemical oxidation of quinoline at the electrodes in the potential range of -0.6 V to +0.6 V. The rise in the magnitude of the Ipa from the bare SPCE as displayed in curve b suggests the impact of the modifiers on the surface of the electrodes. The combined traits of the nanocomposites cannot be over emphasized. The aMWCNTs provide the large surface area, AuNPs are responsible for catalysis, and CTSNPs release the binding potential frameworks. The CV curves in Figure 3d for various bi-layers of nanocomposite modified SPCEs exhibit uneven anodic oxidation peak currents. The Ipa increases from bare/SPCE through BL1/SPCE, attaining an optimal level at BL4/SPCE. Reduction in Ipa is thus observed at BL5/SPCE and BL6/SPCE. The decrease in Ipa may result from the level of thickness of modifiers on the surface of the SPCE which acts as a barrier for rapid electron motion. This implies that electron movement is highly impeded at bi-layers 5 and 6. Thus, BL4/SPCE is recommended for the determination of quinoline [32]. Although, the curve of bare/SPCE may appear to be higher than that of BL1/SPCE (Figure 3d), the actual measurement from the instrument gave a higher peak current value (concrete value) for BL1/SPCE. It is likely that this may have been related to the varying sensitivities of the SPCE. 

The divergent sensitivities of SPCE are known to be a major concern for researchers in this area, so it is not surprising that the bare/SPCE is far more conductive than the one used in modification of the BL1/SPCE. The outcome of cyclic voltammetry peak current determinations of the new bare/SPCE in 1 mM K_3_Fe(CN)_6_/0.1MKCL at 0.05 V/s presented new bare electrodes with closed peak current values which were selected for the modification process. Conclusively, the ultra-thin layer(s) of the nanocomposites utilized in this study caused no harm to the electrode surface.

### 3.3. Effect of Supporting Electrolytes, pH, Accumulation Potential and Time

**Figure 4:**
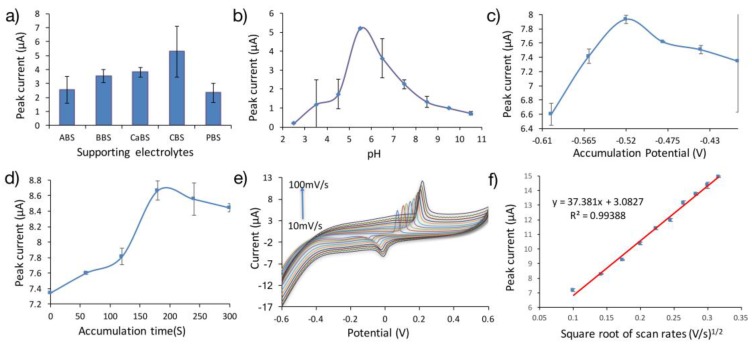
Effect of supporting electrolytes (**a**), pH (**b**), accumulation potential (**c**), and accumulation time (**d**) on the oxidation peak current of 10 μM quinoline. (**e**) Cyclic voltammograms of 10 μM quinoline in 0.2 M CBS (pH 5.5) at BL4/SPCE at various scan rates. (**f**) The relationship of the oxidation peak current (Ipa) and the square root scan rates (v^1/2^).

Supporting electrolytes are often referred to as buffers or buffer solutions. Five different supporting electrolytes of the same concentration namely phosphate (PBS), citrate (CBS), carbonate (CaBS), borate (BBS) and acetate (ABS) buffer solutions were prepared and investigated using 10.0 μM quinoline and BL4/SPCE. The plot for different types of buffer and the oxidation anodic peak current of quinoline is displayed in Figure 4a. Citrate buffer gave the highest anodic oxidation peak current. Therefore, 0.2 M CBS was selected to be used to investigate the remaining electrochemical processes with quinoline. 

The citrate buffer that was chosen as the supporting electrolyte for this study was prepared at varying pH values to cross examine the oxidative current response of quinoline. As depicted in Figure 4b the Ipa increases as the pH value rises to 5.5. Further increase in the pH value to 10.5, gives rise to reduction in the Ipa. Therefore, Ipa in 0.2 M CBS with pH of 5.5 were selected as the analytical values for quinoline detection. This pH value will be applied for the rest of the electro-oxidation reaction of quinoline. 

The electro-oxidation of quinoline was carried out in acidic pH, based on the research of Gupta and Rather (1989) who synthesized quinolinic acid (pyridine-2,3-dicarboxylic acid) from the electro-oxidation of quinoline using 70% sulphuric acid at 60 °C [36]. This condition facilitates the release of protons and electrons from the benzene moiety of the quinoline ring. Also, most of its oxidative reactions take place in the presence of acids. 

The relation between accumulation potentials and anodic oxidation peak current (Ipa) of quinoline at BL4/SPCE in 0.2 M CBS supporting electrolyte was investigated as displayed in Figure 4c. The Ipa of quinoline steadily increased with an increase in accumulation potential from −0.6 V to −0.52 V and a decline with further lead in Ipa. Therefore, maintenance of a high degree of sensitivity requires that an accumulation potential of −0.52 V, which intersects the maximum Ipa, be utilized for the electro-oxidation processes involving quinoline.

The electroactivity of quinoline in 0.2 M CBS at BL4/SPCE with respect to accumulation time in the range 0 s to 360 s was studied and the plot of the outcome is uncovered in Figure 4d. It is evident that Ipa increases with an increase in time until 180 s, while an increase in time leads to a sharp decrease in Ipa. Considering the efficacy and sensitivity of the present analysis, 180 s, which equals the maximum Ipa, is hereby selected as the optimal accumulation time for the electrochemical detection of quinoline.

The effect of the scan rate on the electrochemical response was also surveyed employing cyclic voltammetry. The Ipa of 10 μM quinoline in 0.2 M CBS at the BL4/SPCE increased proportionately with the increase in scan rate (0.01 V/s to 0.1 V/s) (Figure 4e). The linear equation depicting this observed phenomenon was deduced as Ipa (μA) = 37.38v (V/s) + 3.08, with a correlation coefficient (R^2^) of 0.99 (Figure 4f). This linearity implies that the electro-oxidation reaction at the modified electrode is an adsorption controlled process [37] as per the range of scan rate applied. On increasing the scan rate, distortion from linearity surfaced, thus converting the electro-oxidative process to a diffusion-dependent type. The scan rate of 0.06 V/s was accepted for the electrochemical detection of quinoline with a view to ensuring that the entire procedure is confined within the boundary of the adsorption-dependent process.

### 3.4. Repeatability, Reproducibility and Stability.

Under the optimal conditions, we were able to ascertain the extent at which the modified electrodes could be reused and reproduced. The limit of stability was also established. A single BL4/SPCE was evaluated for repeatability by applying it five consecutive times in the electro-oxidation of 10.0 μM quinoline. The calculated relative standard deviation (RSD) of the anodic peak current was 2.94%, corroborating the high level of reusability of the electrode. In addition, five BL4/SPCEs were fabricated separately and employed in the determination of quinoline. The RSD of the anodic peak current (Ipa) on estimation was 3.02%, confirming the fact that the modified electrode is very capable of being reproduced. The BL4/SPCE was under storage at room temperature for twenty (20) days, after which it was used to determine 10 μM quinoline. The bare SPCE was used as a control for the stability study. The modified electrode successfully retained 91.7% of its initial Ipa while the bare SPCE was found to be 40% below the initial Ipa. This large decrease may relate to surface degradation of the bare/SPCE from exposure to the environment and the absence of surface modifiers to retain the current. Conversely, it is likely that the Ipa retention of the modified SPCE relates to the nanocomposite surface modifiers where the CTS provided binding with the analyte, the AuNPs displayed catalytic potential, and the MWCNTs presented a large surface area. We concluded that the combined effect of these nanocomposite attributes contributed to the superior storage stability of the modified electrode.

### 3.5. Interference 

The potential interference for the determination of quinoline was examined under the optimized criteria. The Ipa of quinoline was measured in the vicinity of varying concentrations of interferring species ranging from inorganic to organic and the difference in Ipa was carefully monitored. Near zero counteractive interference was observed in the determination of 1.0 μM quinoline after the inclusion of 1000-fold Cr^3+^, Cu^2+^, Ca^2+^, Ni^2+^. Zn^2+^, Mg^2+^, Fe^2+^, Fe^3+^, l^−^, Cl^−^, and SO_4_^2−^ as well as 500-fold thiourea, 4-dimethyl aminopyrine, urea, histidine, valine, glutamine, acrylamide, phenylalanine, and oxalic acid, as percentage interferences were less than 5%.

### 3.6. Determination of Quinoline Using Linear Sweep Voltammetry and Limit of Detection

**Figure 5:**
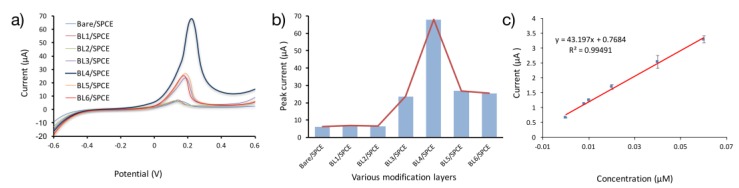
(**a**) Linear sweep voltammetry (LSV) voltammograms of 10 μM quinoline at bare/SPCE, BL1/SPCE, BL2/SPCE, BL5/SPCE, BL6/SPCE, BL3/SPCE, and BL4/SPCE/SPCE in 0.2 M CBS (pH 5.5) at a scan rate of 0.06 V/s (**b**) The peak current of LSV voltammograms at the surface of the electrode with various layers of modification. (**c**) Linear relationship of Oxidation peak current (Ipa) and concentrations.

The electro-oxidation capability of quinoline was tested with linear sweep voltammetry (LSV), a more sensitive method than cyclic voltammetry. Figure 5a shows the LSV curves for the electro-oxidation of 10 μM quinoline in 0.2 M CBS (pH = 5.5). With reference to the optimized conditions (180 s, −0.52 V), an insignificant anodic peak current was observed for Bare SPCE, BL1/SPCE, and BL2/SPCE (Figure 5b). The equivalent peak potential was 0.118 V. This implies largely poor oxidative activity at these electrodes. However, the Ipa increased at the surface of BL3/SPCE, BL5/SPCE, and BL6/SPCE reaching a maximum at BL4/SPCE. This suggests the excellent performance of BL4/SPCE in decreasing the energy barrier needed to actualize the electro-oxidation of quinoline. Deductively, it is obvious that the large increase in the anodic peak current signal at BL4/SPCE is due to the large catalytic surface area of the nanocomposite ultra-thin layers coupled with effective binding power to cumulatively provide high accumulation efficacy to quinoline molecules in the electrochemical process. The newly evolved electrode possesses the required sensitivity mode to detect quinoline.

The BL4/SPCE could selectively detect quinoline. The fact that quinoline is electroactive confirmed this statement. The electrode cannot detect fluoroquinoline. The choice of deploying quinoline as analyte in this study was based on the work of Nusaibah and colleagues (2015), where quinoline and 20 other named metabolites were produced from *G. boninense* infected oil palm roots. The group reported that quinoline was excreted from the oil palm within 24 h of infection by the fungus. Additionally, quinoline was the only metabolite available in the crude extract sample and thus could be detected by the sensor.

The application of LSV under the optimal conditions provided the linear range and limit of detection (LOD). Figure 5c depicts that the anodic peak current (Ipa) of quinoline on BL4/SPCE is proportional to its concentration (μM) from 0.0004–1.0 μM. The linear regression equation was deduced to be Ipa (μA) = 43.197C + 0.7684 (μM) and the correlation coefficient (R^2^) of 0.9949 was calculated, indicating excellent linearity. After 180 s accumulation at −0.52 V potential, the LOD was calculated to be 3.75 nM, utilizing three signal-to-noise ratios.

The linear range of 0.0004–1.0 μM is not clearly visible in the plot as shown in Figure 5c. This was due to the scale employed by the software in plotting the points on the same graph and was overcome by dividing the points into three sets. For simplification, the middle set of data is presented here.

Estimation of LOD used the formula (3 × standard deviation of peak current value (6) from blank solution) / slope of the calibration curve. The bare/SPCE was run in blank solution of 0.2 M CBS for six cycles of CV. The peak current values were obtained and the standard deviation calculated. This value was substituted in the previously mentioned formula for estimating the limit of detection. 

### 3.7. Field Application

The newly developed electrode was tested in the field using real samples sourced from both healthy and infected leaves extracted from oil palms. The recovery study is aimed at evaluating the efficiency of the newly developed electrode. These leaf extracts were confirmed to possess the secondary metabolite called quinoline as well as other defensive secondary metabolites. They were produced in oil palms after 24 h of *G. boninense* attack and present in both healthy and infected standing palms [3]. About 150 mg of powdered leaves of *G. boninense*-infected trees was freeze-dried, then subjected to sonication in 250 mL of 80% stock solution of methanol (30 min and 40 °C). This extractive stage was carried out in two successive sections, filtering the cumulative supernatant through Whatman filter paper (125 mm), and finally, the filtrate was dissolved via a rotary evaporator. The extracts were stored at −80 °C waiting for further analytical procedures. The leaf extracts from the healthy trees were treated using the same approach. For the electrochemical process, a solution of 10 mg leaf extract was produced by adding 0.2 mL methanol, 0.5 mL of 0.2 M CBS (pH = 5.5), and diluting to 10 mL with DI water. The concentrations of quinoline used in the real sample analysis via the standard addition method are listed in Table 1. The LSV curves were recorded from −0.6 V to +0.6 V after 180 s accumulation at −0.52 V. Each sample was determined by three (3) cycles and RSD was lower than 11% (Table 1), suggesting better precision. The Ipa increased with an increase in the amount of quinoline solution added and the peak potential deviated minimally, depicting the real capability of the newly proposed electrode.

Based on the results in Table 1, it would appear that there is more quinoline in the infected leaf extracts than in that of the healthy ones. Above 300% of the added quinoline was recovered from infected samples whereas the percentage was well below 200 in healthy samples, suggesting the production of more quinoline via inducement by the invasion of *G. boninense* on oil palms. The significance of the large amount of quinoline is that it acts as a combatant against the invading pathogenic white rot fungus. This also corroborates the action of quinoline as a defensive agent of oil palm trees. It should be noted that, as a similitude of input and output in mechanical devices, our device measures the difference rather than the content. 

Table 2 shows the comparison of the present work with previous related work, focusing on the metabolite studied, the electrode materials utilized, the type of electroanalytical tool used, the LOD, and the sensitivity of the method. It is clear that, using CV and LSV, the LOD and sensitivity of quinoline detection by this device was superior to comparable devices previously reported.

## 4. Discussion

*G. boninense* continues to cost the oil palm industry vast sums of money as its spreads through plantations. Scientists have tried a number of methods aimed at detecting the disease when it first strikes a plant. The most promising have been electrochemical DNA-based methods, but these are complex because of the difficulty in extraction of infected material from the plant. The method described here overcomes that obstacle by using the leaves of the oil palm and a secondary metabolite, in this case quinoline. It is also possible that this method could be extended by experimenting with other materials such as some of the 2D and layered transition metal oxides reported by Kalantar-zadeh et al. Transition-metal dichalcogenides (TMDs), such as MoS_2_ and WSe_2_, exhibit 2D layered structures identical to that of graphene with good surface-to-volume ratio and conductivity so could be candidates to replace MWCNTs and improve performance. Transition metal oxides are reported to display excellent electron-transfer, have exhibited strong electrocatalytic activity, and have the potential to combine with MWCNTs to form nanohybrids with improved performance [43,44]. 

In the experiment described here, nanomaterials and hybrid nanomaterials were prepared through simple laboratory methods. A novel analytical tool for quinoline was created using the ultra-thin bi-layers of AuNPs-aMWCNTs/CTSNPs-aMWCNTs/SPCE. The AuNPs-aMWCNTs/CTSNPs-aMWCNTs film enhanced the electro-oxidation activity of quinoline, due to the catalytic activity of AuNPs, binding potential of CTSNPs, and large surface area provided by aMWCNTs. The result was excellent signal enhancement of quinoline on AuNPs-aMWCNTs/CTSNPs-aMWCNTs ultra-thin films. In summary, a new sensitive, selective, cost-effective, and environmentally friendly electrode was developed for the detection of quinoline in both healthy and infected leaves of oil palms. It is hoped that this research may be applied in the industry’s battle with *G. boninense*.

## Figures and Tables

**Table 1:** Determination of quinoline in oil palm leaf extracts of healthy (Samples 1, 2, 3) and infected (Samples 4, 5, 6) trees. RSD = relative standard deviation.

	Sample	Added (μM)	Found (μM)	RSD (%)	Recovery (%)
Healthy	1	0.02	0.0343	10.6	171.4
	2	0.04	0.0530	3.6	132.5
	3	0.06	0.0755	4.2	125.8
Infected	4	0.02	0.0651	6.9	325.4
	5	0.04	0.1634	3.5	408.5
	6	0.06	0.1806	1.3	301.0

**Table 2:** Comparison of this work with previous work.

Analyte	Electrode Material	Analytical Method	Linear Range	LOD	References
Shikonin	MWCNTs/β-CD/GCE	CV, DPV	0.005–10.00 μM	0.001 μM	[37]
Mycotoxin (Ochratoxin)	IC nanostructured/AuNPs	DPV	0.3–8.5 ng/mL	0.20 ng/mL	[38]
Phenolics	Au/Lac-CS-MWCNT	CV	0.91–12.1 μM	0.233 μM	[39]
Hydroquinone & catechol	cMWCNT(NHCH_2_CH_2_HN)_n_/GCE	DPV	10–120 μM 5–80 μM	2.3 μM 1.0 μM	[40]
Versicolorin A.	Aptasensor (issAP-Ver.A/Au)	DPV	0.01–100 ng/mL	0.01 ng/mL	[41]
Mycotoxin (Aflatoxins)	IC on MBs/ITO	AMP	0.05–12 ng/mL	0.006 ng/mL	[42]
Alkaloid (Quinoline)	AuNPs-aMWCNTs/CTSNPs-aMWCNTs/SPCE	CV, LSV	0.0004–1.0 μM	3.75 nM	This work

**Abbreviations**: LOD: limit of detection; IC: indirect competitive assay; MBs: magnetic beads; ITO: indium tin oxide electrode; AMP: amperometry; DVP: differential pulse voltammetry; Au: gold; Lac: laccase; CS: chitosan; MWCNT: multi-walled carbon nanotubes; CV: cyclic voltammetry; AuNPs: gold nanoparticles; SPCE: screen printed carbon electrode; LSV: linear sweep voltammetry; SWV: square wave voltammetry; cMWCNT: carboxylated multi-walled carbon nanotubes; GCE: glassy carbon electrode; issAP:-Ver.A: single stranded DNA; β-CD: β-cyclodextrin; CTS: chitosan; aMWCNTs: activated multi-walled carbon nanotubes.
